# Short- and Long-Term Effectiveness of Supplementation with Non-Animal Chondroitin Sulphate on Inflammation, Oxidative Stress and Functional Status in Obese Subjects with Moderate Knee Osteoarthritis before and after Physical Stress: A Randomized, Double-Blind, Placebo-Controlled Trial

**DOI:** 10.3390/antiox9121241

**Published:** 2020-12-07

**Authors:** Mariangela Rondanelli, Niccolò Miraglia, Pietro Putignano, Gabriella Peroni, Milena Anna Faliva, Maurizio Naso, Clara Gasparri, Vittoria Infantino, Mara Nichetti, Nicola Volpi, Federica Capitani, Veronica Mantovani, Simone Perna

**Affiliations:** 1 IRCCS Mondino Foundation, 27100 Pavia, Italy; mariangela.rondanelli@unipv.it; 2 Unit of Human and Clinical Nutrition, Department of Public Health, Experimental and Forensic Medicine, University of Pavia, 27100 Pavia, Italy; viriainfantino@hotmail.it; 3 Clinical & Pre-Clinical Development, Gnosis SpA, 20121 Milan, Italy; miraglia@gnosis.lesaffre.com; 4 SP Diabetic Outpatient Clinic, ASST Monza, 20900 Monza, Italy; pputignano@virgilio.it; 5 Endocrinology and Nutrition Unit, Azienda di Servizi alla Persona “Istituto Santa Margherita”, University of Pavia, 27100 Pavia, Italy; milena.faliva@gmail.com (M.A.F.); mau.na.mn@gmail.com (M.N.); clara.gasparri01@universitadipavia.it (C.G.); dietista.mara.nichetti@gmail.com (M.N.); 6 Department of Life Sciences, University of Modena and Reggio Emilia, 41125 Modena, Italy; nicola.volpi@unimore.it (N.V.); federica.capitani@unimore.it (F.C.); 7 Clinical and Experimental Medicine PhD Program, University of Modena and Reggio Emilia, 41125 Modena, Italy; veronica.mantovani@unimore.it; 8 Department of Biology, College of Science, University of Bahrain, Sakhir Campus, Zallaq P.O. Box 32038, Bahrain; simoneperna@hotmail.it

**Keywords:** nonanimal chondroitin sulfate, knee osteoarthritis, pain, inflammation, overweight, obesity

## Abstract

It has recently been demonstrated that chronic supplementation with nonanimal chondroitin sulfate (nonanimal CS) in overweight subjects with knee osteoarthritis (OA) improves the function, pain and inflammation, but there are no studies of its effectiveness in an acute setting. In 48 obese subjects with moderate knee OA, we investigated the effectiveness of nonanimal CS supplementation for eight weeks on the inflammation, functional status, oxidative stress, cartilage catabolism markers, metabolic profile and body composition, by Dual-Energy X-ray Absorptiometry (DXA) at the baseline, after 15 days and at the end of the eight-week study. To evaluate the acute effectiveness on inflammation, 15-min cycle training sessions were done 15 days after the start of the study and at the end. C-reactive protein (CRP) was assayed in blood samples collected before and after the two cycling exercises. The 48 obese subjects (M and F, 20–50 years, body mass index (BMI) 30–35 kg/m^2^) were randomly assigned to an experimental group (N = 24, 600-mg tablet of nonanimal CS/day) or the control group (N = 24, placebo). The between-groups analysis of covariance showed a significant effect on the Western Ontario and McMaster Universities Arthritis index (WOMAC) scale (*p* = 0.000) and CRP (*p* = 0.022). For intra-group differences, the result was significant in the CS group for BMI, WOMAC, CRP, total cholesterol and Homeostasis Model Assessment (HOMA). In these obese adults with OA, nonanimal CS improved the inflammation, knee function, metabolic profile and body composition.

## 1. Introduction

Knee osteoarthritis (OA) affects a significant proportion of obese subjects [1,2]. Physical exercise is routinely prescribed in order to lose weight and prevent the onset and deterioration of OA. However, exercise may acutely exacerbate local inflammation, causing pain and discouraging people from walking. Different plasma markers of oxidative stress, inflammation and cartilage turnover have been reported to be increased in obesity, particularly in obese subjects following physical therapy [3,4].

The conservative treatment of OA aims to delay cartilage degeneration, and chondroprotective agents are a valid approach for this purpose. Among commercially available dietary supplements, chondroitin sulfate (CS) has proved a safe and effective adjunctive tool to physical exercise to mitigate pain and reduce inflammatory markers [5,6,7]. It was recommended by the European League against Rheumatism (EULAR) as a Symptomatic low-Acting Drug for OsteoArthritis (SYSADOA) for the treatment of OA of the knee, hip and hand [8,9,10].

It has been demonstrated recently that chronic nonanimal CS supplementation in overweight subjects with knee OA improves the function, pain and inflammation [11], with beneficial effects even after only four weeks of treatment. Nonanimal CS supplementation is safe and sustainable, unlike animal-derived CS. The results in this previous trial aroused interest in the use of nonanimal CS in a different context, such as physical stress-generated OA in obese subjects, and the need to clarify better whether its beneficial effects can be achieved after short-term supplementation. The previous study on nonanimal CS included only C-reactive protein (CRP) blood levels, the most commonly investigated systemic inflammation marker, easy to assess in clinical practice, but it did not assess other important markers of inflammation (such as cytokines) or markers of cartilage catabolism (i.e., collagen type II, CTX-II) or synovium metabolism (i.e., hyaluronic acid) and markers of oxidative stress.

Several in vitro studies illustrate the protective features of CS against oxidative stress [12,13] not only in OA but in other pathologies too—for example, neurodegenerative diseases [14,15].

We therefore investigated the longer-term effectiveness of nonanimal CS supplementation given for eight weeks on inflammation markers (CRP, interleukin (IL)-1beta, IL-6 and tumor necrosis factor (TNF)-alpha); the functional status of the knee (Western Ontario and McMaster Universities Arthritis index (WOMAC); oxidative stress (carbonylated proteins); cartilage catabolism markers (plasma keratan sulfate, cartilage oligomeric matrix protein and hyaluronic acid); metabolic profile (lipid profile and Homeostasis Model Assessment, HOMA) and body composition indicated by Dual-Energy X-ray Absorptiometry (DXA) in obese subjects with moderate knee OA, at the baseline, after 15 days and at the end of the eight-week study.

To evaluate the acute effectiveness of the test product on inflammation, since physiology studies have demonstrated that contracting skeletal muscle acutely produces interleukin-6 (IL-6), an inflammatory protein and biologic precursor of CRP [16], 15 min of cycling were scheduled 15 days after the start of the supplementation and at the end of the study; CRP blood samples were analyzed before and after the two cycling exercises.

## 2. Materials and Methods

This was a single-center, prospective, randomized, double-blind, placebo-controlled clinical trial. It was approved by the ethics committee (1215/11122015) of the Department of Internal Medicine and Medical Therapy of the University of Pavia.

### 2.1. Population

Overweight and obese males and females with OA were recruited through the Metabolic Rehabilitation Division of the Santa Margherita Hospital (Azienda di Servizi alla Persona, Pavia, Department of Public Health, University of Pavia, Pavia, Italy) from August 2019 to the end of March 2020. Participants received information on all aspects of the study and were given a written consent form to be signed prior to participation in the study. Data were gathered before they entered the trial. The potential participants underwent a medical screening, comprising demographic information, medical history, current medications and supplements, vital signs, blood and urine tests and a 12-lead electrocardiogram.

### 2.2. Inclusion/Exclusion Criteria

The inclusion criteria specified sedentary Caucasian males or females, with the following characteristics: overweight/obese individuals (body mass index (BMI) 25–40 kg/m^2^); ages 16–75 years; experiencing mobility impairment, joint discomfort or with established moderate knee OA (classification 1–3 according to the Kellgren and Lawrence scoring system for knee OA) and pain intensity 40–70 mm on a visual analog scale (VAS). Subjects with evidence of heart, kidney or liver disease or other diseases that might affect the results of the trial were excluded. Other exclusion criteria comprised ascertained or presumptive hypersensitivity to the active ingredient tested (CS), excessive smoking, abuse of alcohol, presenting or receiving treatment for rheumatoid arthritis and consumption of nutraceuticals or food supplements containing CS in the two weeks before the start of the study. Participants were instructed to avoid nonsteroidal anti-inflammatory drugs (NSAIDs) and high CS-containing foods (animal cartilage, bones or derivatives such as gelatin), which have known anti-inflammatory and/or analgesic effects. In addition, volunteers were instructed not to consume nutraceuticals or food supplements containing CS during the study.

### 2.3. Study Design

**Figure 1:**
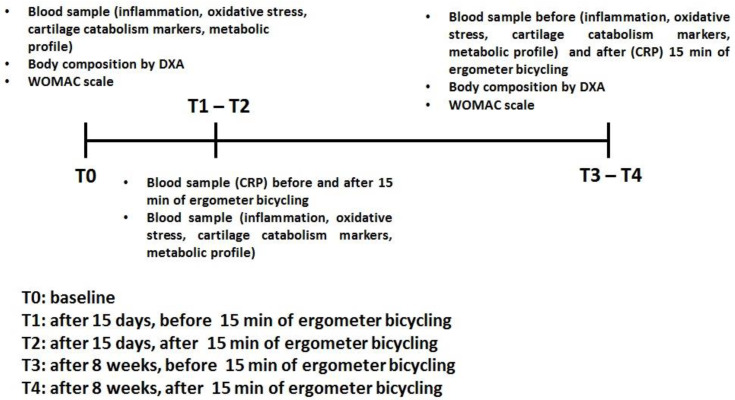
Design of the trial for placebo and experimental groups. WOMAC: Western Ontario and McMaster Universities Arthritis index and CRP: C-reactive protein.

Eligible men and women were randomly assigned to the experimental or the placebo group. The experimental group received 600 mg of Mythocondro^®^ daily for 8 weeks. Mythocondro^®^ was provided by Gnosis by Lesaffre (Desio, Italy), a business unit of the Lesaffre Group (Marcq-en-Baroeul, France). The trial included 15 min of exercise on an ergometer cycle, at 55–60% of their maximum heart rate (calculated from the progressive maximal exercise test), 2 weeks after the beginning of the study (T1-T2) and at 8 weeks (T3-T4). Body composition was measured by DXA and using the WOMAC at baseline (T0) and at 8 weeks (T4). Blood samples for CRP were collected at baseline (T0), after 2 weeks (before, T1 and, after 15 min of ergometer cycling, T2) and at 8 weeks (before, T3 and, after 15 min of cycling, T4). Inflammatory markers (CRP, IL-1beta, IL-6, TNF-alpha and homocysteine); oxidative stress markers (Oxygen Radical Absorbance Capacity (ORAC) test malondialdehyde (MDA) and carbonylated proteins); cartilage catabolism markers (plasma keratan sulfate, cartilage oligomeric matrix protein and hyaluronic acid); metabolic profile (glucose and insulin plasma levels) and the Homeostasis Model Assessment (HOMA) rating were all recorded at baseline (T0), after 15 days (T1) and at 8 weeks (T4). Figure 1 describes the trial design with the experimental and placebo groups.

### 2.4. Description of the Intervention

Mythocondro^®^ is a nonanimal CS produced by chemical sulfation of a nonsulfated chondroitin backbone obtained by thermo-acid hydrolysis of the capsular polysaccharide naturally produced by a specific strain of *Escherichia coli* (O5:K4:H4 strain U1-41(ATCC23502)) [11,17,18,19]. The test product was presented in the form of a 1.02-g tablet, containing 600 mg of Mythocondro^®^ and excipients: microcrystalline cellulose, mannitol, stearic acid, polyvinylpyrrolidone (PVP), silicon dioxide, sodium croscarmellose and magnesium stearate. Participants were asked to swallow one tablet with a glass of water, daily, for 8 weeks. The placebo consisted of tablets (1.02 g) of identical flavor and appearance as the test product. Placebo tablets contained the same excipients of the test tablets lacking the active ingredient. Each participant was instructed to consume one test or placebo tablet per day for 8 weeks.

### 2.5. Tolerance of the Test Product

Tolerance of the nonanimal CS was established on the basis of the absence of side effects, i.e., gastrointestinal symptoms such as nausea and diarrhea. A registered dietician questioned participants by phone daily about any side effects.

### 2.6. Effectiveness of the Test Product

The primary endpoint of the study was the evaluation of inflammatory markers (CRP, IL-1beta, IL-6 and TNF-alpha). The secondary endpoints were the evaluation of: (1) oxidative stress markers (carbonylated proteins); (2) cartilage catabolism markers (plasma keratan sulfate, cartilage oligomeric matrix protein and hyaluronic acid); (3) insulin sensitivity markers (glucose, plasma insulin levels and HOMA index) and (4) knee function (WOMAC) [20].

### 2.7. Pain and Knee Function

The WOMAC pain scale consists of five questions to assess pain during everyday activities. The answers are recorded on a five-point Likert scale, with a higher score indicating a greater level of pain. This scale is validated and reliable in hip and knee OA populations [1]. WOMAC measures five items for pain (scores 0–20), two for stiffness (scores 0–8) and 17 for functional limitation (scores 0–68). Questions about physical functioning cover everyday activities such as use of stairs, standing up from a sitting or lying position, standing, bending, walking, getting in and out of a car, shopping, putting on or taking off socks, lying in bed, getting in or out of a bath, sitting and heavy and light household duties [20,21].

### 2.8. Biochemical Parameters

Blood samples were obtained through an indwelling catheter in an antecubital vein to avoid venipuncture stress. Samples were soon centrifuged and stored at −80 °C pending the analysis. Fasting blood glucose (FBG), total cholesterol (TC), low-density lipoprotein-cholesterol (LDL-C), high-density lipoprotein-cholesterol (HDL-C) and triglyceride (TG) levels were measured with an automatic biochemical analyzer (Hitachi 747, Tokyo, Japan). Serum hemoglobin A1c (HbA1c) was determined by high-performance liquid chromatography using an automatic HbA1c analyzer (Tosoh HLC-723G7, Tosoh Corporation, Tokyo, Japan). Insulin was measured in a double-antibody radioimmunoassay (RIA) (Kabi Pharmacia Diagnostics AB, Uppsala, Sweden) and expressed as pmol/L. The intra- and inter-assay coefficients of the variations were below 6%, and the low limit of detection was 10.7 pmol/L.

To determine insulin resistance, subjects were instructed to fast for 12 h before the blood sample was taken. Insulin resistance was checked using the HOMA [22]. Plasma homocysteine was determined using Architect Ci 4100 d’ABOTT. Intra- and inter-assay coefficients of variation were <4% for all assays. Hematological parameters (red blood cells (RBC), hemoglobin (Hb), hematocrit (HCT), liver enzymes, creatinine and glycemia) were recorded in a multichannel automated blood cell analyzer (Beckman Coulter Gen system-2, Coulter T540, Brea, CA, USA). Plasma folate and vitamin B12 concentrations were measured by the Molloy method with microbiological assays (chloramphenicol-resistant strain and folic acid calibrator) [23].

A high-sensitive (hCRP) enzyme-linked immunosorbent assay (ELISA) from Demeditec Diagnostics GmbH (Kiel, Germany), in vitro diagnostic (IVD) kit Ref. DE740001, was used for CRP. Il-1beta was assayed with an ELISA kit Lot. 0514F0134 from Sigma-Aldrich (St. Louis, MO, USA); IL-6 was tested with an ELISA IVD kit Ref. DE4640 from Demeditec Diagnostics GmbH (Kiel, Germany), and TNF-alpha was measured with an ELISA IVD kit Ref. K9610 from Immundiagnostik AG (Bensheim, Germany).

For the oxidative stress markers, the OxiSelectTM total antioxidant capacity (TAC) ELISA kit Ref. STA-360 from Cell Biolabs Inc. (San Diego, CA, USA) was used; MDA was assayed with an ELISA kit, Cat. E-EL-0060 from Elabscience Biotechnology Inc. (Wuhan, China) and carbonylated proteins with an ELISA kit Lot. 5A30K08300 from Sigma-Aldrich (St. Louis, MO, USA). Plasmatic keratan sulfate (KS) was tested with an ELISA kit Cat. MBS029190 from MyBioSource (San Diego, CA, USA); cartilage oligomeric matrix protein (Human COMP) was measured with an ELISA, Cat. E-EL-H0654 from Elabscience Biotechnology Inc. (Wuhan, China) and hyaluronic acid (HA) with an ELISA kit, Cat. EHH0036 from ABclonal (Woburn, MA, USA).

### 2.9. Body Composition

Fat-free mass and fat mass were measured at t0 and at the end of the study by DXA (Lunar Prodigy, GE Medical Systems, Waukesha, WI, USA). Visceral adipose tissue (VAT) was assessed by DXA Prodigy enCORE software (version 17; GE Healthcare, Chicago, IL, USA), and VAT volume was calculated using a constant correction factor (0.94 g/cm^3^). The software automatically places a quadrilateral box representing the android region outlined by the iliac crest and with an upper height equivalent to 20% of the distance from the top of the iliac crest to the base of the skull [24].

### 2.10. Ergometer Cycling

Fifteen minutes of ergometer cycling were carried out two weeks after baseline and at the end of the study at 8 weeks. The day on which the subjects did the cycling test, they arrived at our laboratory at the same time (9 a.m.) after 48 h without having taken part in any vigorous physical activity and after an overnight fast. The subjects were instructed to eat a standardized meal the evening before the test (380 kcal: 19.7% protein, 55% carbohydrates, 25% fat and 1.3% dietary fiber). Two weeks after the start of the trial, venous serum samples were collected before (T1) and after 15 min of cycling (T2). With the same schedule, 8 weeks after the start of the study, venous serum was collected before (T3) and after (T4) a 15-min cycling session.

The subjects cycled for 15 min at 55–60% of their maximum heart rate (calculated from a progressive maximal exercise test).

### 2.11. Adverse Events

Patients were monitored for any potential gastrointestinal side effects related to the consumption of the test product (common adverse events). Any unexpected adverse event was also recorded.

### 2.12. Run-in, Randomization and Masking

On completion of the baseline assessment, participants were provided with an 8-week supply of experimental or placebo study tablets (blinded to contents). All participants were randomly assigned (1:1) to consume two tablets of nonanimal CS or matching placebo once-daily.

### 2.13. Statistical Analysis

The sample size, determined following the study by Rondanelli, M (2019) [11] as 48 patients (24 in each group), was set with a difference of −0.14 mg/dL of CRP (95% CI −0.26; −0.04), two-sided two-sample *t*-test at 0.05 level of significance, dropout rate 10% and 90% power.

Statistical analysis and reporting of the study were in accordance with the CONSORT guidelines, with the primary analysis based on the full set. For the baseline variables, summary statistics employed frequencies and proportions for categorical data and mean and SD for continuous variables. Baseline variables were compared using chi-square or Fisher’s exact test for categorical outcomes and unpaired *t*-tests for continuous variables, as appropriate.

In the primary analysis, the baseline-adjusted means and 95% confidence interval (CI) given by analysis of covariance (ANCOVA) with the intra-group changes from baseline to 8 weeks were compared for the CS and control groups. The comparisons were adjusted for age and BMI. The effect of the mean differences in the intervention group were correlated with Spearman’s correlation, with each outcome analyzed pre-post-analysis. Adverse events were examined by safety analysis and Fisher’s exact test. The effect of treatment in this “acute” setting was assessed at each follow-up with the analysis of repeated measures between groups, adjusting for age and weight.

All *p*-values were two-sided. *p* < 0.05 was considered statistically significant. All analyses were done with SPSS Version 22.00 (Armonk, NY, USA).

## 3. Results

In total, 48 patients (22 males and 26 females) were enrolled and randomly assigned to either the nonanimal CS (24 patients) or placebo (24 patients). None of the participants refused to take the supplement, and no side effects were reported. The primary and secondary outcomes were analyzed in the full analysis set. Baseline clinical characteristics were similar in the two groups (Table 1), except for BMI, which differed significantly (*p* = 0.031).

Table 2 shows the results of the primary and secondary outcomes. Supplementation with the nonanimal CS resulted in larger reductions in (mean differences and 95% CI) weight (kg) −2.23 (−3.57; −0.89), BMI (kg/m^2^) −0.73 (−1.21; −0.25), WOMAC −8.83 (−14.62; −3.04), CRP (mg/dL) −0.33 (−0.54; −0.12), total cholesterol (mg/dL) −9.77 (−17.75; −1.78), Homeostatic Model Assessment of insulin resistance (HOMA-IR) −0.77 (−1.47; −0.07) and gamma-glutamyltransferase (GGT) (U/L) −3.91 (−7.50; −0.31)*. The between-groups analysis of covariance (intervention minus placebo) showed a significant effect on the WOMAC scale of −22.06 points (−31.79; −12.33) (*p* = 0.000) and of −0.40 on CRP (−0.75; −0.06) (*p* = 0.022). There were no differences between the two groups in any of the other outcomes.

**Figure 2:**
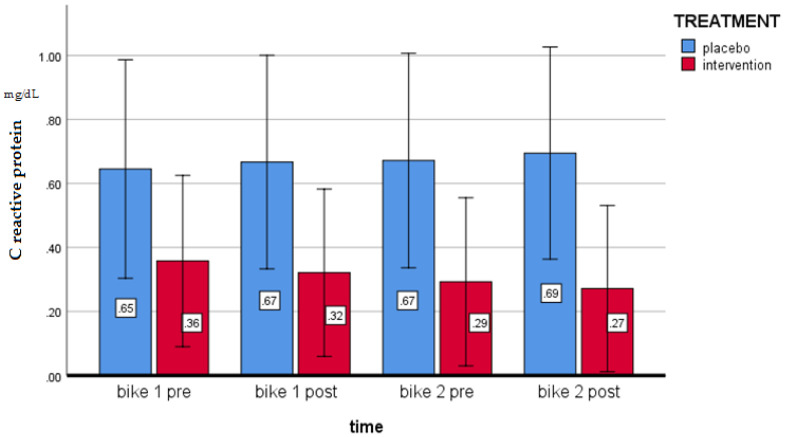
Effects on C-reactive protein of the intervention and placebo during the supplementation period in the “acute” condition—15 min “endurance” cycling (two separate sessions) (*p* = ns).

Figure 2 shows the effects on C-reactive proteins of the intervention and placebo during the supplementation.

A pairwise comparison shows the pre-post (intervention minus placebo) mean changes (between groups and during BIKE1) of −0.058 (95% CI −0.18; 0.71, *p* = ns) after the first cycle of −0.045 (95% CI −0.16; 0.71, *p* = ns) in cycle 2 (between groups and during BIKE2). The multivariate test shows no effects between the two cycles (*p* = 0.485)

**Figure 3:**
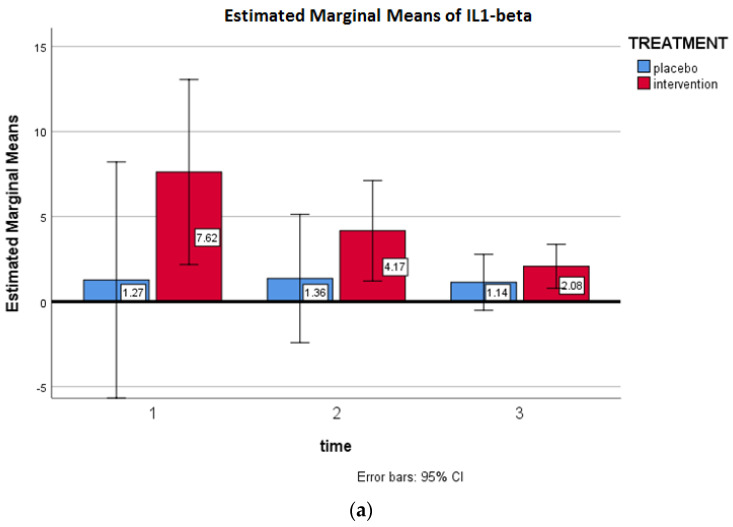

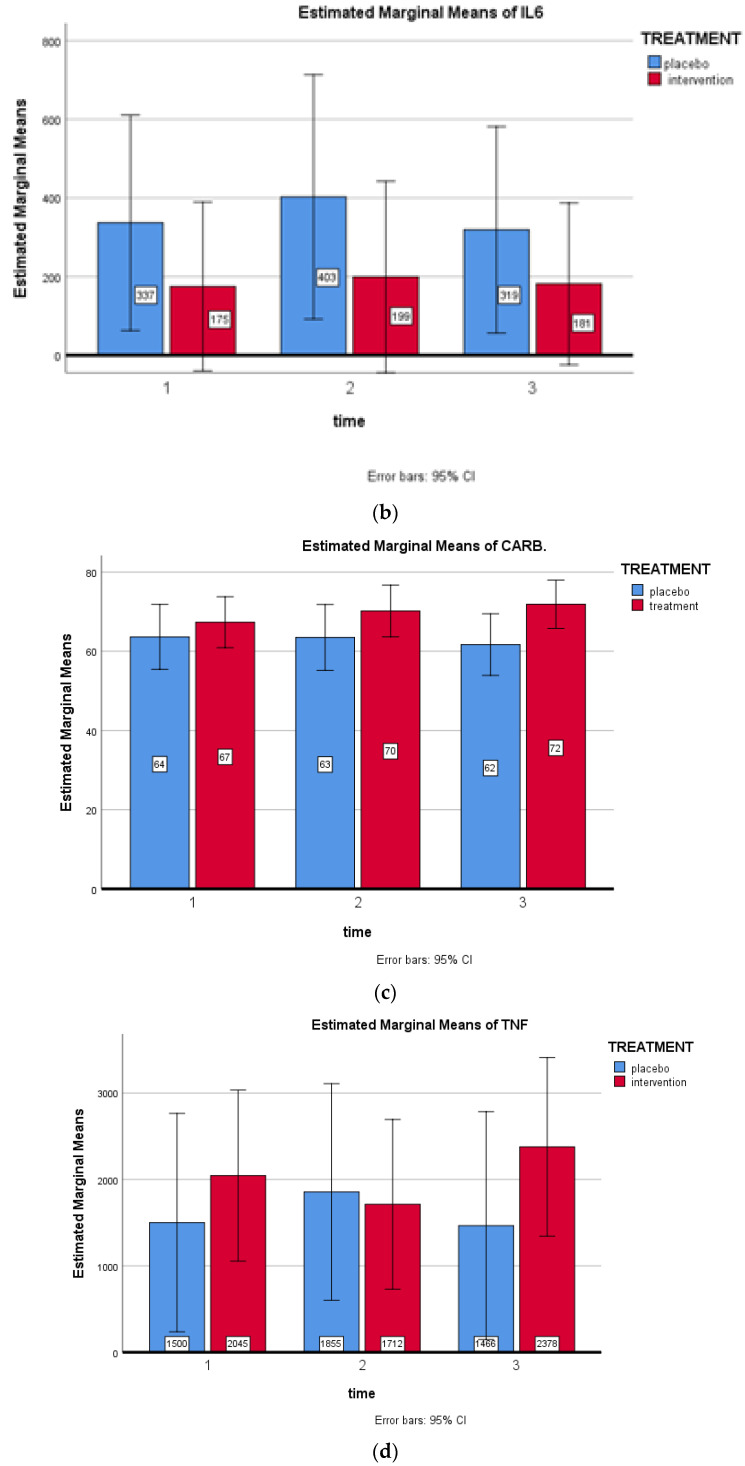

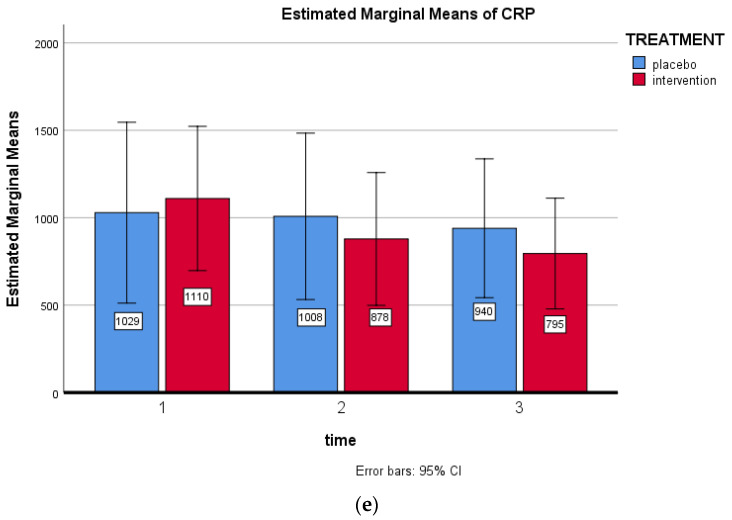
(**a**) Changes in interleukin (IL) 1-beta between the groups over time. (**b**) Changes in IL6 between the groups over time. (**c**) Changes in carbonylated proteins between the groups over time. (**d**) Changes in TNF between the groups over time. (**e**) Changes in CRP between the groups over time.

Figure 3 shows the multivariate analysis for treatment for IL1-beta, IL6, TNF, carbohydrates and CRP. Figure 3a shows the changes in IL1-beta between groups over time. A multivariate analysis adjusted for age and baseline BMI did not show any effects of the treatment compared to placebo (*p* = 0.515). IL1 beta in the intervention group decreased from 7.62 to 2.08, while, in the placebo group, the decrease was from 1.27 to 1.14 mg/dL.

Figure 3b shows the changes in IL6 between groups over time. A multivariate analysis adjusted for age and BMI baseline did not show any effects of the treatment compared to the placebo (*p* = 0.291). IL6 beta in the intervention group increased slightly, from 175 to 181, and in the placebo group, the decrease was from 337 to 319 mg/dL.

Figure 3c shows the changes in carbonylated proteins between the groups over time. A multivariate analysis adjusted for age and baseline BMI did not show any effects of the treatment compared to the placebo (*p* = 0.515). The CARB increased from 67 to 72 in the intervention group, while, in the placebo group, it decreased from 64 to 62 mg/dL.

Figure 3d shows the changes in TNF between the groups over time. A multivariate analysis adjusted for age and the baseline BMI did not show any effects of the treatment compared to the placebo (*p* = 100). TNF-alpha in the intervention group increased from 2045 to 2378, while, in the placebo group, it decreased from 1500 to 1466 mg/dL.

Figure 3e shows the changes in CRP between the groups over time. A multivariate analysis adjusted for age and baseline BMI showed no effects of the treatment compared to the placebo.

Table 3 reports a Spearman’s correlation analysis of Δchanges (t1–t0). There were no significant changes. Some suggested associations (but not statistically significant) were reported: patients whose BMI decreased had an improvement in glucose (*r* = 0.303). Patients whose glucose improved also showed an improvement on the WOMAC scale (*r* = −0.146).

All these associations need further investigation in order to calculate the *p*-value.

## 4. Discussion

This randomized, double-blind, placebo-controlled trial illustrated the beneficial effects of an eight-week supplementation with nonanimal CS on the functional status and inflammation in obese patients with moderate OA compared to a placebo. The effect of this CS was assessed considering several parameters related to inflammation (plasma CRP and IL-1beta, IL-6 and TNF-alpha); function; WOMAC and oxidative stress (carbonylated proteins). The decreases in CRP and WOMAC ratings in the CS-supplemented group indicate a beneficial effect of the supplementation and confirm the findings of a previous study [11].

To test the efficacy of this nonanimal CS supplement in an acute inflammatory manifestation such as physical stress, since physiology studies have shown that contracting skeletal muscle acutely produces interleukin-6 (IL-6), an inflammatory protein and an inducer of CRP expression [16], some of the parameters were analyzed before and after a cycling session. As regards the efficacy of this CS supplementation after physical activity, there was a trend—though not significant—toward a reduction of inflammation, indicated by CRP, only in the treated group. This reduction of acute CRP, even though not significant, might demonstrate some effect of nonanimal CS in counterbalancing the low-grade inflammation usually seen with obesity [25].

One very interesting finding is the significant reductions in BMI, total cholesterol and HOMA in the subjects supplemented with the nonanimal CS but not in the placebo group after eight weeks. This is quite likely related to the increase in daily activities, reflecting the analgesic effect of the nonanimal CS. However, one can also hypothesize some effect of the dietary supplement on metabolism. These findings have not been previously reported in humans, although an antiobesity effect of chondroitin sulfate was demonstrated in vitro and in an animal model, with chondroitin sulfate of animal origin (skate CS) [26]. In addition, Han et al. showed that salmon nasal CS inhibited lipase activity in vitro and that oral treatment resulted in less fat storage in mice fed a high-fat diet [27]. The other parameters examined did not change significantly either in or between the groups.

We investigated cartilage catabolism markers and oxidative stress in order to gain a better understanding of the activity of nonanimal CS, but these oxidative stress-related parameters showed no significant changes. Chondroitin sulfate was shown to reduce the molecular damage caused by free radicals and associated oxygen reactants [28].

CS is reported to achieve its antioxidant activity through the upregulation of Nrf2, along with endogenous antioxidants; it reduces apoptosis by inhibiting the mitochondrial pathway to protect SH-SY5Y cells damaged by 6-hydroxydopamine (6-OHDA) [15]. However, contradictory data have also been reported in human studies [29,30].

The nonsignificant changes of cartilage catabolism markers and oxidative stress in this trial might be due to the low sample size and to the fact that these parameters have wide biological variability [31,32]. However, the small number of subjects is the main limitation.

## 5. Conclusions

In conclusion, a nonanimal CS supplementation in obese adults with moderate OA improved the inflammation and knee function in a short time, confirming a previous study. This CS supplementation also improved metabolic profiles and body composition, raising interest in a fuller assessment of the utility of nonanimal CS in the treatment of obesity. Finally, how nonanimal CS counteracts oxidative stress remains to be clarified.

## Figures and Tables

**Table 1:** Clinical characteristics of the sample at the baseline.

Variable	Control (24, 11 M, 13 F) Mean; SD	Intervention (24, 11 M, 13 F) Mean; SD	Total Sample (48)	*p*-Value between Groups
Age (years)	43.36 ± 8.67	48.73 ± 16.33	46.64 ± 13.97	0.267
Height (m)	1.68 ± 0.11	1.65 ± 0.14	1.66 ± 0.13	0.481
Weight (kg)	95.38 ± 16.15	88.482 ± 18.84	91.16 ± 17.93	0.267
BMI (kg/m^2^)	33.47 ± 1.85	32.09 ± 1.77	32.63 ± 1.91	0.031
FFM (kg)	51.43 ± 9.43	49.61 ± 10.88	50.32 ± 10.24	0.611
FM (kg)	41.27 ± 10.12	36.13 ± 10.72	38.13 ± 10.65	0.162
FM (%)	44.29 ± 6.47	41.82 ± 6.36	42.78 ± 6.43	0.268
VAT (kg)	1.77 ± 0.92	1.40 ± 1.17	1.55 ± 1.08	0.327
WOMAC	23.61 ± 16.71	22.19 ± 16.16	22.74 ± 16.15	0.802
CRP (mg/dL)	0.68 ± 0.88	0.65 ± 0.77	0.66 ± 0.80	0.918
Total cholesterol (mg/dL)	192.14 ± 32.39	182.59 ± 40.31	186.31 ± 37.24	0.461
Triglycerides (mg/dL)	117.93 ± 57.91	104.00 ± 51.78	109.42 ± 53.87	0.457
Glucose (mg/dL)	86.71 ± 9.14	86.73 ± 9.96	86.72 ± 9.52	0.997
HDL (mg/dL)	54.36 ± 17.85	52.46 ± 15.93	53.19 ± 16.48	0.741
LDL (mg/dL)	132.29 ± 27.10	121.36 ± 40.01	125.61 ± 35.53	0.376
VLDL (mg/dL)	23.59 ± 11.58	20.80 ± 10.36	21.88 ± 10.77	0.457
Insulin (IU/mL)	12.66 ± 6.78	13.30 ± 9.67	13.05 ± 8.56	0.829
HOMA-IR	2.73 ± 1.50	2.93 ± 2.21	2.85 ± 1.94	0.770
AST (U/l)	18.71 ± 7.75	17.64 ± 4.73	18.06 ± 6.0	0.606
ALT (U/l)	24.07 ± 16.10	19.46 ± 9.28	21.25 ± 12.38	0.282
GGT (U/l)	25.79 ± 16.36	19.86 ± 14.61	22.17 ± 15.36	0.265
Creatinine (mg/dL)	0.79 ± 0.16	0.79 ± 0.16	0.79 ± 0.16	0.921
Vitamin B12 (pg/mL)	362.64 ± 131.25	419.05 ± 205.86	397.11 ± 180.56	0.368
Folic acid (ng/mL)	6.89 ± 6.80	8.06 ± 8.36	7.61 ± 7.71	0.664
Homocysteine (µmol/L)	14.15 ± 3.69	15.30 ± 8.11	14.85 ± 6.70	0.624
WBC (×10^3^/µL)	6.35 ± 1.64	6.37 ± 1.58	6.36 ± 1.58	0.971
Lymphocytes (×10^3^/µL)	1.87 ± 0.41	2.05 ± 0.66	1.98 ± 0.57	0.346
Lymphocytes (%)	30.64 ± 7.90	33.04 ± 8.13	32.11 ± 8.02	0.388
RBC (×10^6^/µL)	4.96 ± 0.38	4.77 ± 0.36	4.84 ± 0.38	0.135
Hemoglobin (g/dL)	14.12 ± 1.98	13.64 ± 1.11	13.83 ± 1.50	0.353
Hematocrit (%)	41.67 ± 3.06	40.81 ± 3.04	41.14 ± 3.03	0.411
MCV (fl)	84.38 ± 7.82	85.95 ± 7.44	85.34 ± 7.52	0.549

In bold type, *p* < 0.05. The values in the table are mean ± standard deviation. Abbreviations: ALT, alanine aminotransferase; AST, aspartate transaminase; BMI, body mass index; CRP, C-reactive protein; FFM, fat-free mass; FM, fat mass; GGT, gamma-glutamyltransferase; HDL, high-density lipoprotein; HOMA-IR, Homeostatic Model Assessment of insulin resistance; LDL, low-density lipoprotein; MCV, mean corpuscular volume; RBC, red blood cells; VAT, visceral adipose tissue; VLDL, very-low-density lipoprotein; WBC, white blood cells and WOMAC, Western Ontario and McMaster Universities Osteoarthritis Index.

**Table 2:** Within- and between-group mean changes from the baseline (from day 0 to the end of the intervention) for clinical markers.

Variable	Control Intra-Group Δ Change (CI 95%)	Intervention Intra-Group Δ Change (CI 95%)	Intervention Effect between Groups *p*-Value
Weight (kg)	−1.12 (−2.83; 0.59)	−2.23 (−3.57; −0.89) **	−1.10 (−3.36; 1.15) (*p* = 0.325)
	−0.40 (−1.02; 0.21)	−0.73 (−1.21; −0.25) **	−0.33 (−1.14; 0.48) (*p* = 0.416)
FFM (kg)	−0.16 (−1.13; 0.81)	−0.32 (−1.09; 0.44)	−0.16 (−1.44; 1.12) (*p* = 0.797)
FM (kg)	−1.13 (−2.57; 0.32)	−1.44 (−2.58; −0.31)	−0.32 (−2.22; 1.59) (*p* = 0.739)
FM (%)	−0.56 (−1.94; 0.83)	−0.63 (−1.72; 0.46)	−0.07 (−1.90; 1.76) (*p* = 0.937)
VAT (kg)	−0.05 (−0.34; 0.24)	−0.16 (−0.39; 0.07)	−0.11 (−0.49; 0.27) (*p* = 0.552)
WOMAC	13.23 (5.85; 20.62)	−8.83 (−14.62; −3.04) *	−22.06 (−31.79; −12.33) (*p* = 0.000)
CRP (mg/dL)	0.28 (−0.23; 0.29)	−0.38 (−0.58; −0.17) *	−0.40 (−0.75; −0.06) (*p* = 0.022)
Total cholesterol (mg/dL)	−0.92 (−9.28; 11.11)	−9.77 (−17.75; −1.78) **	−10.68 (−24.11; 2.75) (*p* = 0.115)
Triglycerides (mg/dL)	−1.44 (−22.09; 19.20)	−5.40 (−21.57; 10.78)	−3.95 (−31.16; 23.25) (*p* = 0.769)
Glucose (mg/dL)	3.42 (−3.04; 9.87)	−3.13 (−8.18; 1.93)	−6.54 (−15.05; 1.96) (*p* = 0.127)
HDL (mg/dL)	2.84 (−3.67; 9.35)	2.05 (−3.05; 7.15)	−0.79 (−9.37: 7.79) (*p* = 0.852)
LDL (mg/dL)	−0.11 (−10.07; 9.85)	−5.93 (−13.74; 1.87)	−5.83 (−18.95; 7.30) (*p* = 0.373)
VLDL (mg/dL)	−0.29 (−4.42; 3.84)	−1.08 (−4.32; 2.16)	−0.79 (−6.23; 4.65) (*p* = 0.769)
Insulin (IU/mL)	−0.37 (−4.08; 3.35)	−2.87 (−5.78; 0.04)	−2.50 (−7.40; 2.39) (*p* = 0.306)
HOMA–IR	−0.01 (−0.91; 0.89)	−0.77 (−1.47; −0.07) **	−0.76 (−1.94; 0.42) (*p* = 0.200)
AST (U/L)	−0.41 (−3.95; 3.13)	−0.03 (−2.74; 2.81)	0.44 (−4.23; 5.10) (*p* = 0.850)
ALT (U/L)	0.54 (−4.59; 5.66)	−2.30 (−6.31; 1.72)	−2.83 (−9.58; 3.92) (*p* = 0.399)
GGT (U/l)	−1.08 (−5.66; 3.51)	−3.91 (−7.50; −0.31) **	−2.83 (−8.88; 3.21) (*p* = 0.347)
Creatinine (mg/dL)	−0.03 (−0.07; 0.01)	0.02 (−0.02; 0.05)	0.05 (−0.01; 0.10) (*p* = 0.099)
Vitamin B12 (pg/mL)	14.67 (−42.50; 71.84)	8.26 (−36.54; 53.06)	−6.41 (−81.75; 68.93) (*p* = 0.864)
Folic acid (ng/mL)	−0.15 (−4.42; 4.12)	1.31 (−2.03; 4.66)	1.46 (−4.16: 7.09) (*p* = 0.600)
Homocysteine (µmol/L)	1.97 (−11.47; 15.42)	3.66 (−6.88; 14.19)	1.69 (−16.03; 19.40) (*p* = 0.848)
WBC (×10^3^/µL)	−0.37 (−1.27; 0.52)	0.01 (−0.69; 0.71)	0.39 (−0.80; 1.57) (*p* = 0.511)
Lymphocytes (×10^3^/µL)	0.03 (−0.17; 0.24)	−0.02 (−0.18; 0.15)	−0.05 (−0.32; 0.22) (*p* = 0.719)
Lymphocytes (%)	2.31 (−2.28; 6.90)	−0.28 (−3.87; 3.32)	−2.59 (−8.63; 3.46) (*p* = 0.389)
RBC (×10^6^/µL)	0.02 (−0.09; 0.12)	0.05 (−0.358; 0.13)	0.03 (−0.11; 0.17) (*p* = 0.668)
Hemoglobin (g/dL)	−0.29 (−0.91; 0.33)	0.19 (−0.30; 0.67)	0.48 (−0.34; 1.29) (*p* = 0.243)
Hematocrit (%)	4.70 (0.00; 9.40) **	−0.09 (−3.77; 3.59)	−4.79 (−10.98; 1.40) (*p* = 0.125)
MCV (fl)	−4.96 (−10.75; 0.83)	1.08 (−3.46; 5.61)	6.04 (−1.59; 13.67) (*p* = 0.117)

* The values in the table are mean ± standard deviation. Abbreviations: ALT, alanine aminotransferase; AST, aspartate transaminase; BMI, body mass index; CRP, C-reactive protein; FFM, free fat mass; FM, fat mass; GGT, gamma-glutamyltransferase; HDL, high-density lipoprotein; HOMA-IR, Homeostatic Model Assessment for insulin resistance; LDL, low-density lipoprotein; MCV, mean corpuscular volume; RBC, red blood cells; VAT, visceral adipose tissue; VLDL, very-low-density lipoprotein; WBC, white blood cells and WOMAC, Western Ontario and McMaster Universities Osteoarthritis Index. ** *p* < 0.05.

**Table 3:** Spearman’s correlation analysis of Δchanges (t1–t0) among all other significant outcomes in the intervention group.

VARIABLES		ΔWOMAC	ΔHOMA	ΔChol	ΔGlucose	ΔBMI
ΔWOMAC	Correlation Coefficient	1.000	0.027	−0.128	−0.147	0.010
	Sig. (2-tailed)	0	0.906	0.570	0.513	0.966
ΔHOMA	Correlation Coefficient	0.027	1.000	−0.100	−0.080	0.286
	Sig. (2-tailed)	0.906	0	0.659	0.724	0.197
ΔChol	Correlation Coefficient	−0.128	−0.100	1.000	0.149	−0.023
	Sig. (2-tailed)	0.570	0.659	0	0.507	0.919
ΔGlucose	Correlation Coefficient	−0.147	−0.080	0.149	1.000	0.303
	Sig. (2-tailed)	0.513	0.724	0.507	.	0.170
ΔBMI	Correlation Coefficient	0.010	0.286	−0.023	0.303	1.000
	Sig. (2-tailed)	0.966	0.197	0.919	0.170	0
